# IL6 pretreatment promotes chemosensitivity by eliminating quiescent cancer (stem) cells in lung adenocarcinoma

**DOI:** 10.1002/ctm2.217

**Published:** 2020-10-31

**Authors:** Xin Wang, Xiaotong Zhao, Lin Shi, Yuan Wu, Xiaomei Zhang, Zhaohui Fan, Bo Shen

**Affiliations:** ^1^ Department of Internal Oncology Jiangsu Cancer Hospital (Nanjing Medical University Affiliated Cancer Hospital) and Jiangsu Institute of Cancer Research Nanjing China; ^2^ Medical Department III for Hematology and Oncology, School of Medicine Klinikum rechts der Isar, Technical University of Munich Munich Germany; ^3^ Department of Otolaryngology Head and Neck Surgery Affiliated Hospital of XuZhou Medical University Xuzhou China; ^4^ Charité‐Universitätsmedizin Berlin Corporate Member of Freie Universität Berlin Humboldt‐Universität zu Berlin Berlin Germany; ^5^ Department of Thoracic Surgery Jiangsu Cancer Hospital (Nanjing Medical University Affiliated Cancer Hospital) and Jiangsu Institute of Cancer Research Nanjing China

Dear Editor,

Lung cancer is one of the most devastating malignancy globally. Despite great development of target and immunotherapies, cytotoxic chemotherapy is still regarded as the backbone for lung cancer treatment. Clonal heterogeneity and selection pressure from chemotherapy together contribute to chemoresistance.^1^ Cancer stem cells (CSCs) are a subpopulation of cancer cells that have the capabilities of self‐renewability, tumor initiation, and therapeutic resistance. Cell quiescence is defined as one of the hallmarks of CSCs^2^; thus, elimination of quiescent tumor cells provides a practical approach for cancer treatment, aiming to lower the chances of recurrence (or at least extends the remission time).^3^ IL6 pretreatment promotes entry into cell cycle, which significantly reverses the chemo‐resistant by reducing quiescent cancer (stem) cells in Cisplatin (cDDP) chemotherapy. The effect of IL6 pretreatment depends on the existence of TFAP2A.

Lung adenocarcinoma tissues were collected from surgical resections for primary lung cancer cell (PLCC) preparation. The PPLCs were stained with CSC surface markers and sorted to CSCs (CD133^+^/CD44^+^/CD24^−^), non‐CSCs (CD133^−^/CD44^−^/CD24^+^), and control (full populations, sorted without selection) subpopulations^4^, ^5^, ^6^, ^7^, ^8^ (Figure 1A). The CSC subgroup showed accumulation of oligopotential cell population as measured by an increase in the number of colony formation units, (Figure 1B, Figure S1). Also, CSCs have the advantage of forming bigger dense colonies (Figure 1C).

**FIGURE 1:**
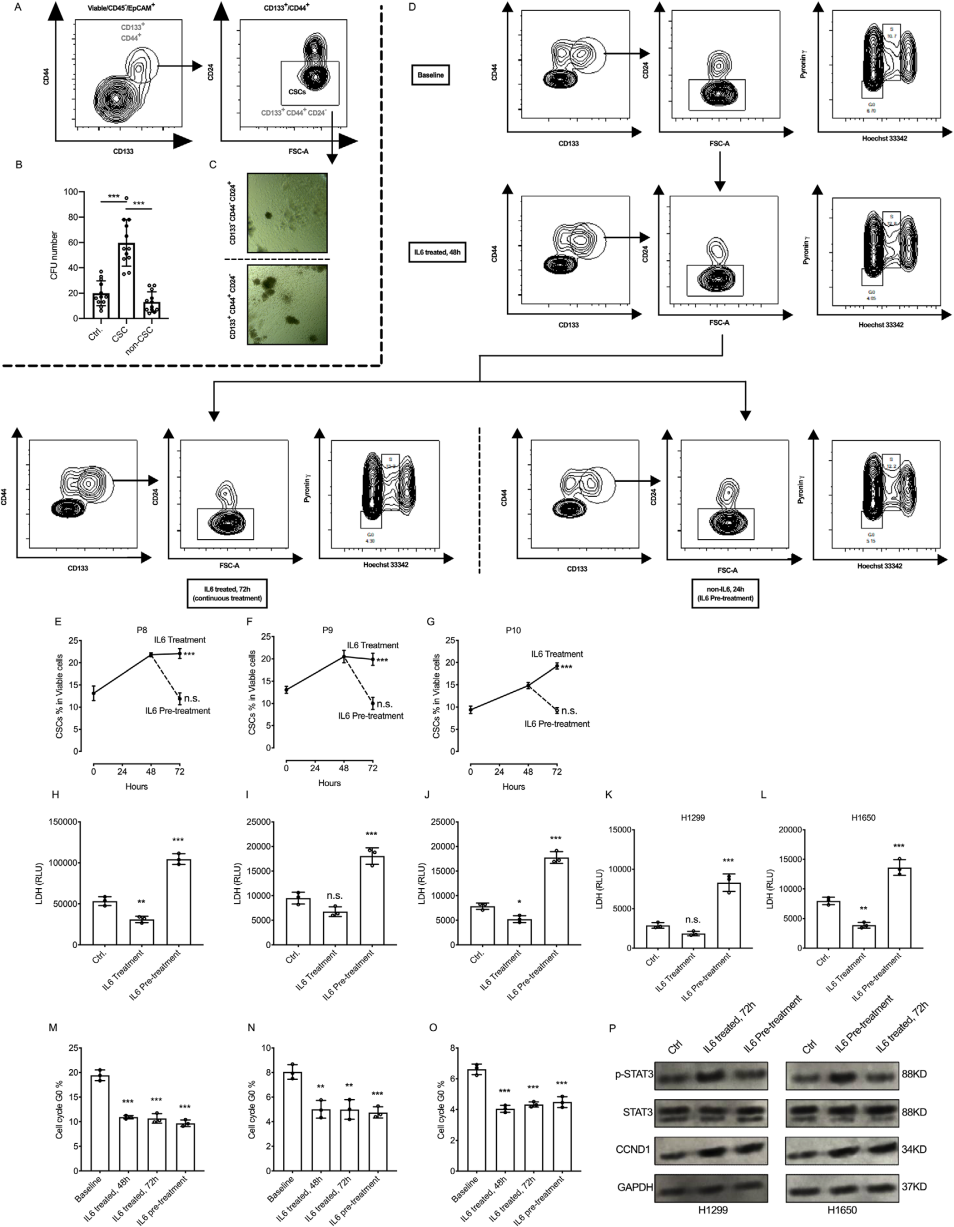
The activated IL6‐STAT3 pathway (but not cell cycle pathway) closes rapidly upon the loss of IL6 signaling and contributes to the chemosensitivity in IL6 pretreatment. A, FACS and sorting strategy for lung cancer stem cells; CD133^+^, CD44^+^, and CD24^−^ lung cancer cells (P1, P3, P4, and P7) were gated and sorted as cancer stem cells (CSCs). B, Primary lung cancer cells were sorted to CSCs (CD133^+^/CD44^+^/CD24^−^), non‐CSCs (CD133^−^/CD44^−^/CD24^+^) and control (cells sorted without selection) subgroups for colony formation assay in Matrigel. Compared with control and non‐CSC group, CSC groups have the advantage of forming more colonies. CFU, colony formation unit. C, CD133^+^, CD44^+^, and CD24^−^ cancer stem cells were seeded in Matrigel for colony formation assay, the example pictures showed the morphology difference of colonies originated from cancer stem cells or non‐cancer stem cells. Cancer stem cells have the advantage of forming bigger dense colonies. D, The FACS plots and FACS flowchart of primary lung cancer cells (PLCC), the cells were treated with IL6 (2 ng/ml) for 48 hours, after that lung cancer cells were cultured in medium with or without IL6 (2 ng/ml) for another 24 hours. Then, cells were collected for CSC staining and cell cycle staining. E‐G, The percentages of CSC population were measured at baseline, at 48 hours and 72 hours. Compared to baseline, in primary lung cancer cells (P8, P9, and P10) at 48 hours, the CSC population were expanded. At 72 hours, the CSC population remain expanded (or kept expanding in P10) in IL6 continues treatment groups, while the percentages of CSC population decreased rapidly (to the baseline level) in IL6 pretreatment groups. H‐L, The primary lung cancer cells (P8, P9, and P10) and lung cancer cell lines (H1299 and H1650) were treated with IL6 (2 ng/mL) for 48 hours, then reseeded with same cell number, after that cDDP was added to the medium (5 μM final concentration) with or without IL6 (2 ng/mL) for 24 hours (for control group, no IL6 was added for 72 hours), next the supernatants were collected for cell death measurements. We found the continues IL6 treatment protect lung cancer cells from death during chemotherapy, but IL6 pretreatment contributes to the chemosensitivity compared to the control group. M‐O, Same experimental settings to (B‐D), and the percentages of G0 population were measured at baseline, at 48 hours and 72 hours. Compared to the baseline, the IL6 treatment significantly decreased the G0 populations, and upon the loss of IL6 signaling, the G0 population remained similar to 48 hours group. P, The western blots of STAT3 pathway and cell cycle indicator (cyclin D1) in H1299 and H1650 cells, (Control group), cells without treatment for 72 hours. (IL6 treated, 72 hours group), Cells treated with IL6 (2 ng/mL) for 72 hours. (IL6 pretreatment group), Cells treated with IL6 (2 ng/mL) for the first 48 hours, then fresh medium was changed for the next 24 hours. The protein samples were harvested for western blot, our data indicated the rapidly close of STAT3 pathway without continuous IL6 treatment, but interestingly, the CCND1 level remains high in IL6 pretreatment group compared to the baseline level

In Figures S2‐S5, the percentage of CSCs, cell cycle distribution, and cell death were measured, and we found that the IL6 pretreatment contributed to the expansion of CSCs and also increased the chemosensitivity by eliminating G0 cells. Thus, the biological differences between continuously fed IL6 and IL6 pretreatment for chemotherapy were further explored. PLCCs were collected for measuring the CSC percentage. Cell cycle distribution staining at different time points after 48 hours of IL6 treatment showed expansion of CSCs, and G0 population was decreased in all PLCCs. After 72 hours, the CSC populations were increasing (P10) or stayed similar (P8 and P9), but more interestingly, upon the loss of IL6 treatment, the percentage of CSCs was quickly decreased to the baseline level (Figure 1E‐G), but not the G0 population (Figure 1M‐O). Moreover, western blotting of IL6‐STAT3 pathway and cell cycle indicator cyclin D1 indicated that IL6 treatment has potently activated the IL6‐STAT3 signaling pathway, but loss of IL6 signaling led to the decrease of p‐STAT3 levels to the baseline level, but not cyclin D1 levels (Figure 1P). The other groups of cells were reseeded with same cell number and treated with IL6 for 48 hours. Next, cDDP (final concentration 5 μM) was added for chemotherapy with or without IL6 for 24 hours followed by LDH cell death measurement (Figure 1H‐L). It is interesting to note that IL6 pretreatment elevated the cytotoxic effects of cDDP chemotherapy (Figure 1H‐L). Our data indicated that loss of external IL6 signaling has rapidly closed the CSC pathway (as well as IL6‐STAT3 pathway) but not activated cell cycle pathway, contributing to the cDDP chemotherapy.

In Figure S6A and B, TFAP2A^KD^ cells were introduced, and TFAP2A was passively sensitive to IL6 (pre)treatment in lung cancer cells. TFAP2A transcription factor binds to AP2 motif and contributes to aggressive phenotypes in malignant lung disease.^9^, ^10^ By analyzing three TFAP2A ChIP‐Seq GEO datasets, ChIP enrichment peaks were identified at the core promoter region of IL6R (Figure 2A). As shown in ChIP‐qPCR, the TFAP2A pull‐down has significantly enriched the DNA fragments from IL6R promoter region (Figure S7A). The dual‐luciferase reporter assay (Figure S7B) and the qPCR (Figure S7C) data further confirmed this result. After that, the wild‐type and TFAP2A^KD^ lung cancer cells were treated with recombinant hIL6 in serial concentration for 48 hours. The results revealed that the TFAP2A^KD^ has significantly decreased the protein levels of IL6R but not STAT3, and the serial IL6 treatment potently activated the IL6‐STAT3 signaling pathway, resulting in a dose‐dependent activation of p‐STAT3 and elevated CCND1 level. Moreover, the IL6R levels were lower, and less STAT3 was activated in TFAP2A^KD^ group, indicating the restriction of IL6‐STAT3 pathway in TFAP2A^KD^ lung cancer cells (Figure 2B). Collectively, TFAP2A has promoted the activation of IL6‐STAT3 pathway after IL6 treatment by transcriptionally activating IL6R.

**FIGURE 2:**
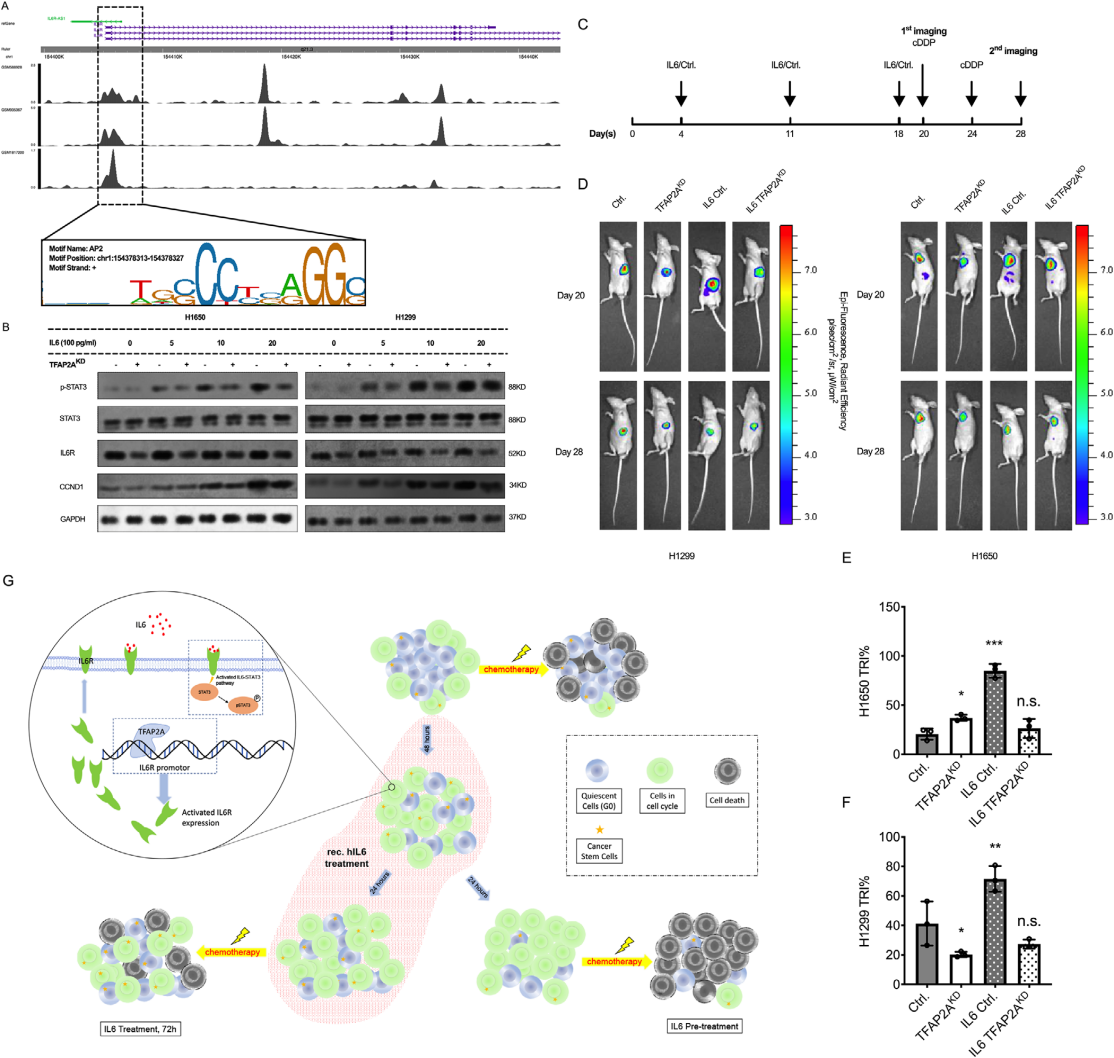
TFAP2A promotes the IL6 sensitivity by promoting the expression of IL6‐Receptor. A, Three independent ChIP‐Seq datasets from GEO were introduced for evaluating the role of TFAP2A on IL6‐STAT3 pathway. TFAP2A pull‐down ChIP‐Seq peaks were identified on the core promoter region of IL6R (GSM588928, GSM935367, and GSM1817200), indicating the potential transcriptional activation for the IL6R expression exists. B, The western blot of IL6‐STAT3 and cell cycle marker. H1650 and H1299 wildtype (WT) or TFAP2A^KD^ cells were treated with IL6 in serial concentrations for 48 hours, after that cells were collected for p‐STAT3, STAT3, CCND1, and IL6R western blot, GAPDH served as reference protein. TFAP2A^KD^ significantly decreased the protein levels of IL6R, and the serial concentration IL6 treatment activated the IL6‐STAT3 pathway, which potently induced the activation of p‐STAT3, and increased CCND1 level (indicated the entering into cell cycle). C, Timeline of in vivo experiment, WT or TFAP2A^KD^, H1299 and H1650 lung cancer cells expressing luciferase were tail‐vein injected to BALB/c nude mice, and IL6 was administrated at day 4, 11, and 18, at day 20 the first bioluminescence imaging was performed, followed by cisplatin administration, and at day 28 the second bioluminescence imaging was performed. D, the bioluminescence image of mice at day 20 and day 28. E and F, Tumor Regression Index (TRI) was introduced for evaluating the tumor extinction rate after chemotherapy, TRI was evaluated as (first fluorescence intensity − second fluorescence intensity)/first fluorescence intensity, shown are the TRI after cDDP chemotherapy at day 28. In line with our ex‐vivo data, the in‐vivo data indicated that, IL6 pretreatment contributed to the cDDP chemosensitivity, and TFAP2A^KD^ undermined the biological effects of IL6 pretreatment

Finally, animal model was introduced to evaluate the impact of IL6 pretreatment and TFAP2A^KD^ in cDDP chemotherapy (tail vein). On day 20, the first imaging followed by cisplatin administration was performed, and the second bioluminescence imaging was performed on day 28 (Figure 2C,D). Compared with WT group, TFAP2A^KD^ group showed additional gaining of tumor burden after significant reduction of IL6 administration, indicating the existence of a restricting effect to the sensitivity of IL6 in lung cancer cells (Figure S8B and C). Based on the data from second imaging, the tumor regression index (TRI) was evaluated by the formula (first tumor burden − second tumor burden)/first tumor burden, which reflects the rate of tumor extinction after chemotherapy. Similar to our ex vivo and in vitro data, IL6 pretreatment in WT mice has significantly strengthened the cytotoxic effects of cDDP chemotherapy, resulting in increased TRIs (Figure 2E and F). Also restricting the role with regard to the effectiveness of IL6 pretreatment was also observed in TFAP2A^KD^ mice, and the results showed that the reduction of TRI was limited in TFAP2A^KD^ IL6 pretreated mice (compared with WT IL6 pretreated mice) (Figure 2E and F). Moreover, the Immunohistochemistry (IHC) staining of cleaved caspase 3 by harvesting lungs of mice indicated that IL6 pretreatment increased the apoptosis rate, while TFAP2A^KD^ restricted this effect (Figure S8A), and this was in line with the TRI data. In summary, our data indicated that the IL6 treatment in vivo has expanded the tumor burden, and IL6 pretreatment resulted in effective killing following cDDP chemotherapy. Moreover, TFAP2A^KD^ restricted the biological effects of IL6 (pre)treatment.

We reported that IL6 pretreatment assisted in entering into the cell cycle in quiescent lung cancer (stem) cells and significantly increased the chemosensitivity. In parallel, the biological effect of IL6 pretreatment depends on the existence of TFAP2A, and TFAP2A in turn regulates the expression of IL6R transcriptionally.

## CONFLICT OF INTEREST

The authors declare that there is no conflict of interest that could be perceived as prejudicing the impartiality of the research reported.

### ETHICS APPROVAL AND CONSENT TO PARTICIPATE

Ethical consent was approved by the Committees for Ethical Review of Research involving human subjects at Jiangsu cancer hospital. The animal experiments were approved by the Use Committee for Animal Care at Nanjing medical university.

### AUTHOR CONTRIBUTIONS

Bo Shen and Xin Wang participated in the study design. Xin Wang and Xiaotong Zhao conducted the in vitro, ex‐vivo, and in vivo experiment. Lin Shi and Xiaomei Zhang conducted the data analysis. Zhaohui Fan provided the human samples. Xin Wang, Bo Shen, and Yuan Wu wrote the manuscript. All the authors read and approved the final manuscript.

## Supporting information

SUPPORTING INFORMATIONS

## Data Availability

All the datasets used in the paper are cited with Gene Expression Omnibus Accession Number. All the other data generated in this study are included in the article.
