# Supplementary material for: IL6 pretreatment promotes chemosensitivity by eliminating quiescent cancer (stem) cells in lung adenocarcinoma
Source: Clin Transl Med. 2020 Oct 31;10(6):e217. doi: 10.1002/ctm2.217 (PMC7603296; doi:10.1002/ctm2.217)
Supplement: Supplementary file 1 — SUPPORTING INFORMATIONS [file CTM2-10-e217-s001.pdf]

Supplementary material

IL6 pre-treatment promotes chemosensitivity by eliminating quiescent cancer (stem) cells in lung adenocarcinoma

Running title, IL6 pre-treatment promotes chemosensitivity

*Xin Wang[1, 2], *Xiaotong Zhao[3, 4], Lin Shi[1], Yuan Wu[1], Xiaomei Zhang[1], Zhaohui Fan[5], #Bo Shen[1]

*these authors contribute equally to this work.
#corresponding author

1. Department of internal oncology, Jiangsu cancer hospital (Nanjing medical university affiliated cancer hospital) and Jiangsu institute of cancer research
2. Technical university of Munich (TUM), Klinikum rechts der Isar
3. Department of otolaryngology head and neck surgery, Affiliated hospital of XuZhou medical university
4. Charité-Universitätsmedizin Berlin, Corporate Member of Freie Universität Berlin, Humboldt-Universität zu Berlin
5. Department of thoracic surgery, Jiangsu cancer hospital (Nanjing medical university affiliated cancer hospital) and Jiangsu institute of cancer research

#Corresponding Author, Bo Shen,

Department of internal oncology, Jiangsu cancer hospital (Nanjing medical university affiliated cancer hospital) and Jiangsu institute of cancer research

Postal address, Department of internal oncology
Jiangsu cancer hospital
Baiziting 42
210009; Nanjing; Jiangsu; China

Tel., (+86) 25-8328-3554; Fax., (+86) 25-8328-3597
Email, shenbo987@njmu.edu.cn

Methods

Tumor sample processing and *in-vivo* experiment

Fresh tumor resections were collected and transported on ice immediately after surgical removal from lung adenocarcinoma (LUAD) patients, upon arrival the tumor tissues were washed with sterile PBS containing 2% FBS, normocin (Invitrogen, 1:200) and antibiotic-antimycotic (Invitrogen, 1:50) for 30 min at room temperature on an orbital shaker. Then, the tumor tissue was diced to small pieces (sesame size) and digested with enzymatic digestion buffer containing liberase (Roche, 0.25mg/ml) and DNase (Sigma, 0.25mg/ml) in serum free RPMI1640 for 45 min at a 37 °C themoshaker. After that, cell suspensions were pipetted vigorously to confirm complete dissociation and passed through a 70 μm cell strainer. For the first 48 hours, primary lung cancer cells were cultured in DMEM / F-12 medium supplemented with 10% FBS, cytokine mix (1 μg/ml insulin, 10 ng/ml EGF), antibiotic mix (1:200, normocin and 1:50, antibiotic-antimycotic) and ROCKi (10 μM, ROCK inhibitor, only for the first 48 hours); followed with low concentration geneticin (G418, 50 mg/ml) treatment for another 48 hours to remove fibroblasts. Next, primary cells were cultured for another 48 hours with fresh medium for stabilization. Animal experiment was conducted following NIH animal use guidelines and protocols approved by Nanjing Medical University Animal Care Committee. 24 BALB/c nude mice at 6-8 weeks old were obtained from Nanjing Medical University affiliated animal facility; after washing, the luciferase expression H1299 and H1650 cells suspended in PBS were tail vein injected (200μl PBS with $5x10^6$ cells per mice and 3 mice per group); and rec. hIL6 (50 mg/kg) was intraperitoneally administrated every 7 days starting from day 3; After 20 days, the first bioluminescence imaging was performed and analyzed with IVIS Lumina LT Series III imaging system; Then, the cisplatin was intraperitoneally administrated (5 mg/kg, twice); 4 days after last cisplatin administration, the second bioluminescence imaging was performed. Details regarding timeline are shown in Figure 2C.

Flow cytometry analysis

For FACS staining, cells were washed and resuspended at the concentration of 1×10^6 cells / ml in FACS buffer (2% FBS in PBS with 0.5mM EDTA). Then cells were pre-incubated with Fc-block for 30 min and subsequently stained with FACS antibodies (CD133, EMK08, Thermo Fisher; CD24, ML5, BioLegend; CD44, #53289, CST; EpCAM, #158206 R&D; CD45, Invitrogen, HI30; IL6R, ab128008, Abcam, all in 1:200 dilution) for 2 hours at 4 °C. PI (Propidium iodide) staining (PI was added before loading the samples to flow cytometer) was used for viability gating. For cell cycle distribution assay, cells were harvested and fixed in 80% ice cold ethanol for 30min, after that cells were washed with PBS twice followed by Hoechst 33342 (Sigma) and Pyronin y (Sigma) staining. Briefly, cells were first stained with Hoechst 33342 (10μg/ml) at room temperature for 30 min followed with Pyronin y (1μg/ml, added directly in the FACS buffer with Hoechst 33342) at room temperature for 20 min. For supernatant IL6 measurement the CBA (cytokine beads array) were introduced according to the official instruction (Human IL6 Flex Set, 558276, Becton, Dickinson and Company). Flow analysis and cell sorting were performed on a FACSAria II flow cytometer (BD Biosciences). Data were analyzed using FlowJo™ (version 10.2; FlowJo LLC). Each assay was performed in triplicate.

Cells, culture conditions, plasmids transduction, CCK8, wound healing and colony formation assay

H1650 cells and H1299 cells were obtained from American Type Culture Collection (ATCC, USA). Cells were cultured in RPMI1640 medium (Thermo Fisher Scientific, Waltham, USA) supplemented with 10% fetal bovine serum and 1% penicillin / streptomycin (Thermo Fisher Scientific, Waltham, USA) and cultured at 37°C in a humidified incubator containing 5% CO_2. For cytokine treatment, rec. hIL6 (P05231, R&D, 2ng/ml), rec. hEGF (236-EG, R&D, 1ng/ml), rec.

hIL3 (203-IL, R&D, 10ng/ml), rec. hCCL2 (279-MC, R&D, 20ng/ml), rec. hCCL5 (335-RM, R&D, 50ng/ml) and rec. hTGF-ß (240-B/CF, R&D, 1ng/ml) were added in the medium for 48 hours. The human TFAP2A shRNA targeting 5'-AACATCCCAGATCAAACTGTA-3' was adopted from the published paper [1], and inserted into pGFP-C-shLenti vector for lentivirus packaging. The Lenti-luciferase-P2A-Neo plasmid was introduced for generating luciferase expressing cells. After that, the H1650 and H1299 cells were transduced with packaged lentivirus. Three days after transduction, for TFAP2A knockdown cells ($TFAP2A^{KD}$), the GFP positive cells were sorted for further culture. And for luciferase expression cells, geneticin (G418, 400μg/ml) was added in the medium for 7 days. The luciferase reporter plasmids (firefly luciferase) covering the promotor region of IL6R was customized from RiboBio (Guangzhou, China); Besides, a Renilla luciferase plasmid reported previously was introduced as background signal [2, 3]. The luciferase activity measurement was described before [2, 3]. For proliferation measurement, the CCK8 kit (KGA317, KeyGen Biotech) was introduced according to the official instruction, and the 450 nm absorbance was measured with a plate reader (Promega, GM3000). For wound healing assay, in 6 well plates artificial scratches on a monolayer of cells were created using a 200μl pipette tip, after that cells were imaged at baseline and 36 hours. For colony formation assay, all triplicate cultures were performed in Matrixgel based on RPMI1640 or DMEM media supplemented with 10% fetal bovine serum and cytokine mix for primary cell culture, lung cancer cells were sorted and seeded at a density of 2000 cells/plate. After 10-14 days, the colonies were counted by light microscopy.

Western blot, qRT-PCR, ChIP-qPCR, IHC staining and LDH measurement

For cell lysis, the cells were treated in RIPA buffer supplemented with complete protease inhibitor cocktail (Roche), protein phosphatase inhibitors sodium orthovanadate and sodium fluoride (Sigma-Aldrich) on ice for 1 hour. After centrifugalization, the proteins were collected from supernatant. For protein quantification, the Pierce BCA Protein Assay (Thermo Scientific) was used following official instruction. Next, the same amount of protein in Laemmli (15 μg) was loaded on gels and separated by 12% SDS-PAGE gels and then transferred to nitrocellulose membranes (GE Healthcare). The following antibodies were used for protein detection: TFAP2A (Abcam, ab52222, 1:1000), pSTAT3 (ab76315, Abcam, 1:2000), STAT3 (#9139, CST, 1:1000), IL6R (ab128008, Abcam, 1:300), CCND1 (ab134175, Abcam, 1:5000) and GAPDH (sc-47724, Santa Cruz, 1:500). After incubated with secondary HRP-conjugated antibodies, blots were detected using Pierce ECL Plus Substrate (Thermo Scientific) and a ChemoCam Imager following official instruction. For qRT-PCR, as described before, the total RNA was isolated and reverse transcribed to cDNA, and qPCR was performed with SYBR Select Master Mix (Applied Biosystems, Cat: 4472908) [2]. The primers were as follows: IL6R, forward 3'-CCCCTCAGCAATGTTGTTTGT-5' and reverse 3'-CTCCGGGACTGCTAACTGG-5'. For ChIP-qPCR, the methods were described before [2, 3]. Briefly, the DNA were prepared with EpiTect ChIP OneDay Kit (QIAGEN, Cat No: 334471) and the chromatin shearing was performed using Sonicator 3000 with following conditions: Power: 0.5 watt; Time: 2 seconds ON / 15 seconds OFF; Total time: 16 seconds (8 times per round); 3 rounds. Negative controls were rabbit IgG (#PP64, Millipore, 1:50) and H3K4me3 (Abcam, ab8580, 1:100) pull down served as a positive control (the sequence of EIF4A2 was detected, Forward, 3'-TTTTGTAGCTGACCGAAGCA-5' and Reverse, 3'-GCGCCCTATGACCTTCACTA-5') and IgG pull down as negative control; for TFAP2A pull down, a ChIP grade antibody were used (Abcam, ab52222, 1:50). The primers for IL6R promotor region were as follows: Forward, 3'-TGTGCCTTTACGGTGGTGAG-5' and Reverse, 3'-CAGAAAAGGGGATCCGTCGC-5'. For relative ChIP enrichment calculation, the enrichment of other samples was normalized to the IgG pulldown and $2^{-(CtTarget - CtIgG)}$. For cleaved caspase 3 immunohistochemistry (IHC) staining, the slides were incubated with cleaved caspase 3 antibody (CST, #9661, 1:200) overnight at 4 °C, then sides were incubated using the secondary antibody for 2 hours in room temperature. The staining intensity was scored as 4 grades: 0 (No staining), 1 (weak staining), 2 (moderate staining), or 3 (intense staining). The percentage of positive cells x respective intensity scores was used as the final staining score (from 0 to 300) [3]. The supernatant LDH measurement was introduced for cell death measurement. The supernatants

were collected 24 hours after cDDP treatment and the LDH levels were measured using LDH-Glo™ Cytotoxicity Assay (J2380, Promega) following official instruction with a plate reader (GM3000, Promega), and LDH level was presented as relative luminescence unit (RLU).

Data sources and statistical analysis

The gene set enrichment analysis (GSEA) was performed following official instruction. GEO dataset GSE62941 containing 3 untreated control H1650 samples and 3 anti-IL6 treatment H1650 samples were introduced, and the latest official tool was obtained from http://software.broadinstitute.org/gsea (Ver. 4.0). Data were presented as the mean ± SD (standard deviation) and analyzed using a student's t-test. $P<0.05$ was considered to indicate a statistically significant difference (*$P<0.05$, **$P<0.005$, ***$P<0.0005$). Excel (Office 365, Microsoft) and GraphPad Prism (version 8.4, GraphPad Software, Inc) software were used for statistical analyses and the production of graphs.

Citations

1. Scibetta AG, Wong PP, Chan KV, Canosa M, Hurst HC. Dual association by TFAP2A during activation of the p21cip/CDKN1A promoter. *Cell Cycle*. Nov 15 2010;9(22):4525-32. doi:10.4161/cc.9.22.13746
2. Guoren Z, Zhaohui F, Wei Z, et al. TFAP2A Induced ITPKA Serves as an Oncogene and Interacts with DBN1 in Lung Adenocarcinoma. *Int J Biol Sci*. 2020;16(3):504-514. doi:10.7150/ijbs.40435
3. Yuanhua L, Pudong Q, Wei Z, et al. TFAP2A Induced KRT16 as an Oncogene in Lung Adenocarcinoma via EMT. *Int J Biol Sci*. 2019;15(7):1419-1428. doi:10.7150/ijbs.34076

Supplementary Figures

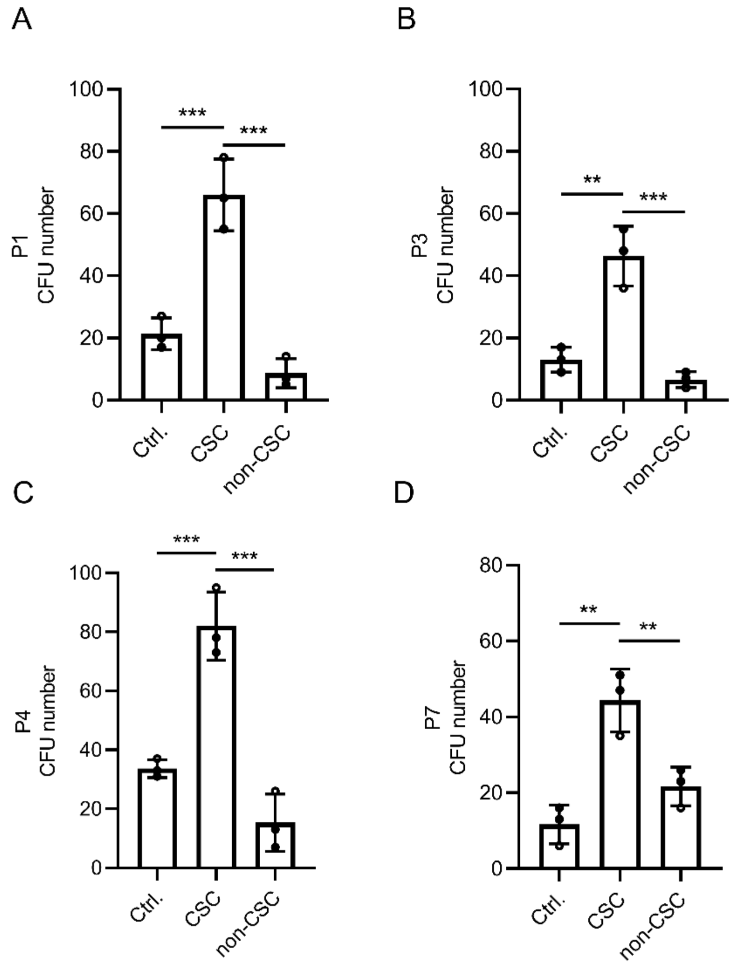

Figure S1, the cancer stem subpopulation in lung cancer cells

A-D, primary lung cancer cells were sorted to CSCs (CD133$^+$/CD44$^+$/CD24$^-$), non-CSCs (CD133$^-$/CD44$^-$/CD24$^+$) and control (cells sorted without selection) subgroups for colony formation assay in Matrixgel. Compared with control and non-CSC group, CSC groups have the advantage of forming colonies.

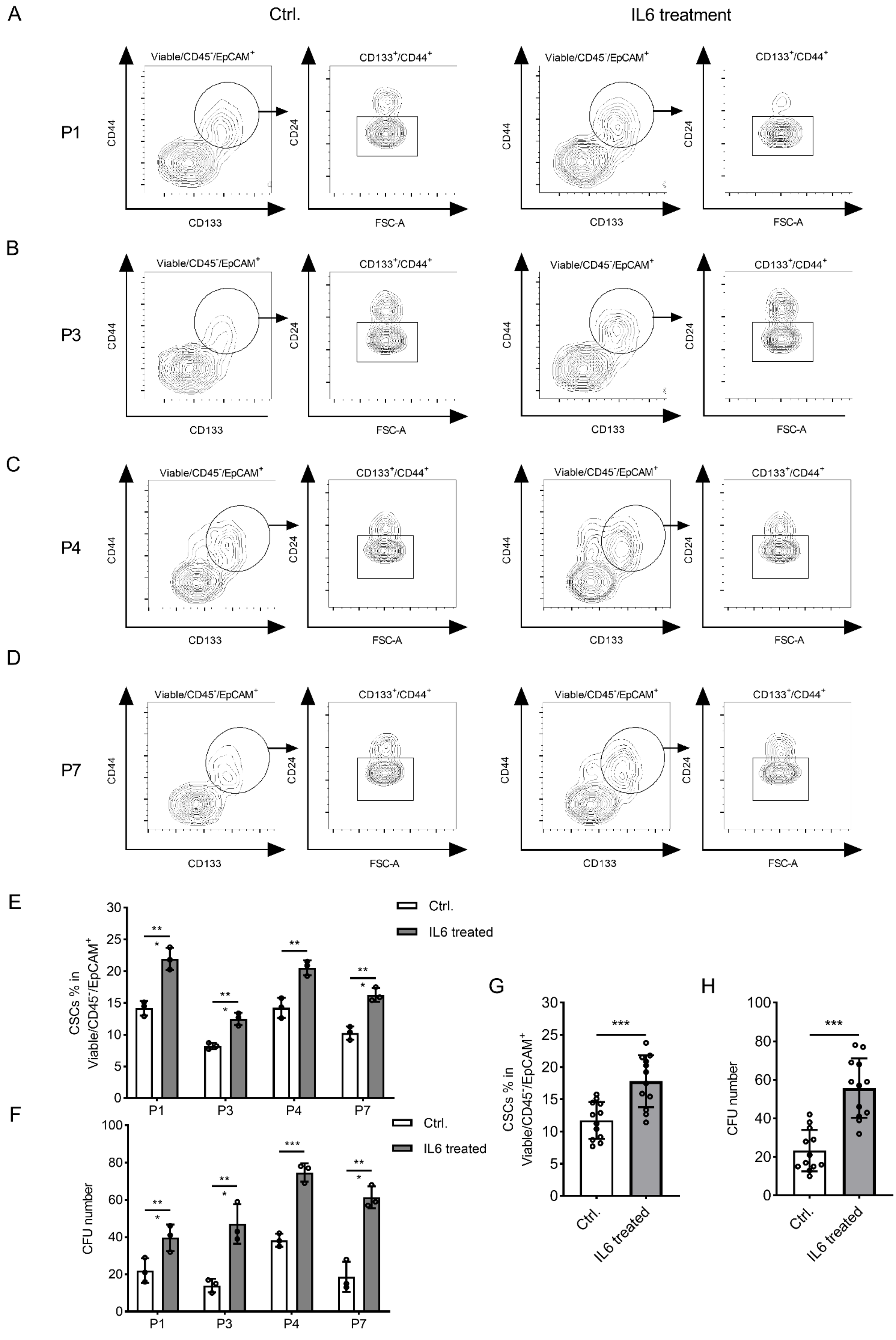
A
Ctrl.
IL6 treatment
P1
Viable/CD45⁻/EpCAM⁺
CD133⁺/CD44⁺
CD44
CD133
CD24
FSC-A
B
P3
C
P4
D
P7
E
CSCs % in Viable/CD45⁻/EpCAM⁺
Ctrl.
IL6 treated
F
CFU number
G
H
P1
P3
P4
P7
Ctrl.
IL6 treated
**
*
***

Figure S2, the impact of IL6 treatment to cancer stem cell distribution

Four primary LUAD cells were treated with or without IL6 for 48 hours, after that cells were measured for cancer stem cells (CSCs) distribution, and CSCs were sorted for colony formation assay.

A-D, the FACS plots for CSCs distribution of patients derived primary lung cancer cells (P1, P3, P4 and P7).

E & G, the percentage of CSCs in primary lung cancer cells when treated with or without IL6. Similar phenotypes were observed cross all primary lung cancer cells, and IL6 treatment significantly expanded the CSC subpopulation

F & H, the colony formation unit (CFU) of sorted CSCs for Matrixgel colony formation assay were counted. IL6 treatment significantly promoted the colony formation ability

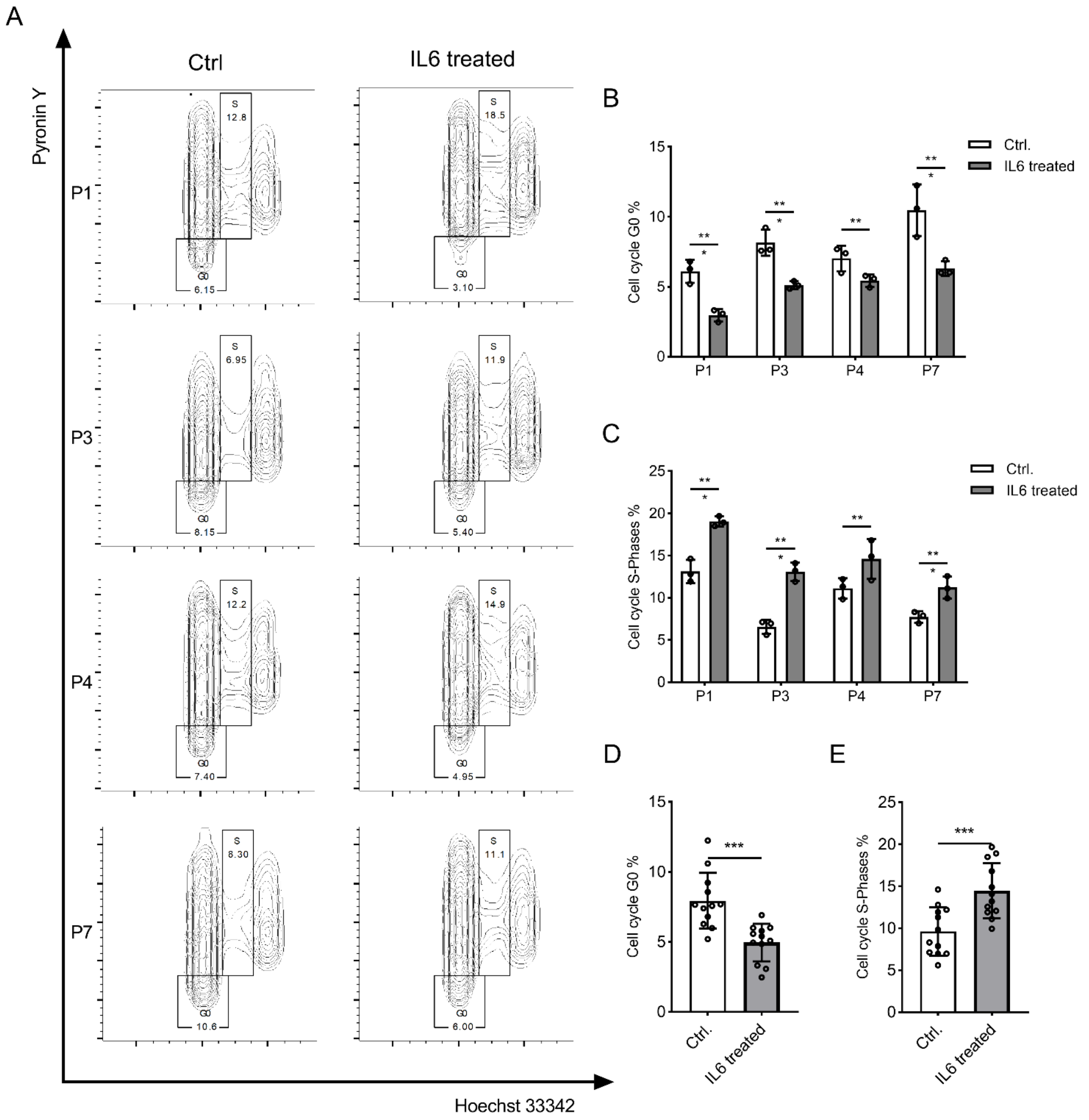

Figure S3, IL6 promotes the entry of cell cycle in quiescent cancer cells

Primary LUAD cells were treated with or without IL6 for 48 hours, after that cell cycle distribution measurement was performed (Hoechst 33342 and Pyronin γ double staining).
A, the gating plots and strategy for cell cycle distribution in Viable/CD45^{-}/EpCAM^{+} primary lung cancer cells, the percentage of cells stay at G0 and S-phase were gated.
B & D, the percentage of cells stay at G0 phase were counted. IL6 treatment significantly decreased the G0 population cross all primary lung cancer cells.
C & E, the percentage of S-phase lung cancer cells were counted. IL6 treatment significantly reduced S-phase populations cross all primary lung cancer cells.
Our data indicated the biological impact of IL6 to cell cycle distribution, that IL6 treatment promoted the entry into cell cycle.

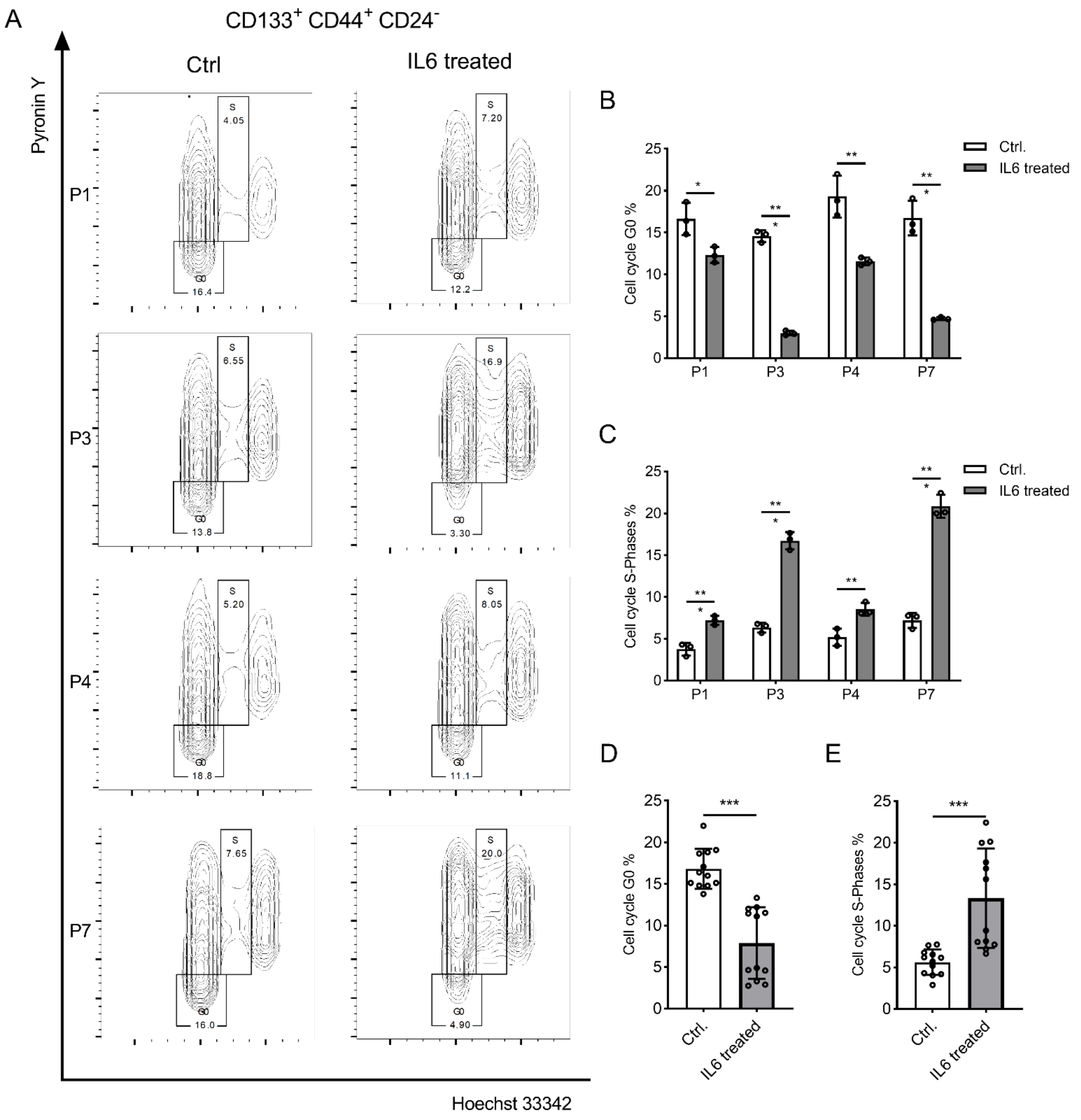

Figure S4, IL6 potently promotes quiescent cancer stem cells to enter cell cycle

Primary LUAD cells were treated with or without IL6 for 48 hours, after that CD133^{+}/CD44^{+}/CD24^{-} cells were sorted for cell cycle distribution measurement (stained with Hoechst 33342 and Pyronin γ).

A, the gating plots and strategy for cell cycle distribution in primary lung cancer stem cells (CSCs), the percentage of cells stay at G0 and S-phase were gated.

B & D, the percentage of CSCs stay at G0 phase were counted. IL6 treatment significantly decreased the G0 population cross all primary lung cancer stem cells.

C & E, the percentage of S-phase lung cancer stem cells were counted. IL6 treatment significantly reduced S-phase populations cross all primary lung cancer stem cells.

IL6 treatment potently promoted the entry into cell cycle (especially P3 and P7) in the cancer stem cell populations ($CD133^{+}/CD44^{+}/CD24^{-}$), expanded S-phase populations and reduced G0-phase CSCs populations.

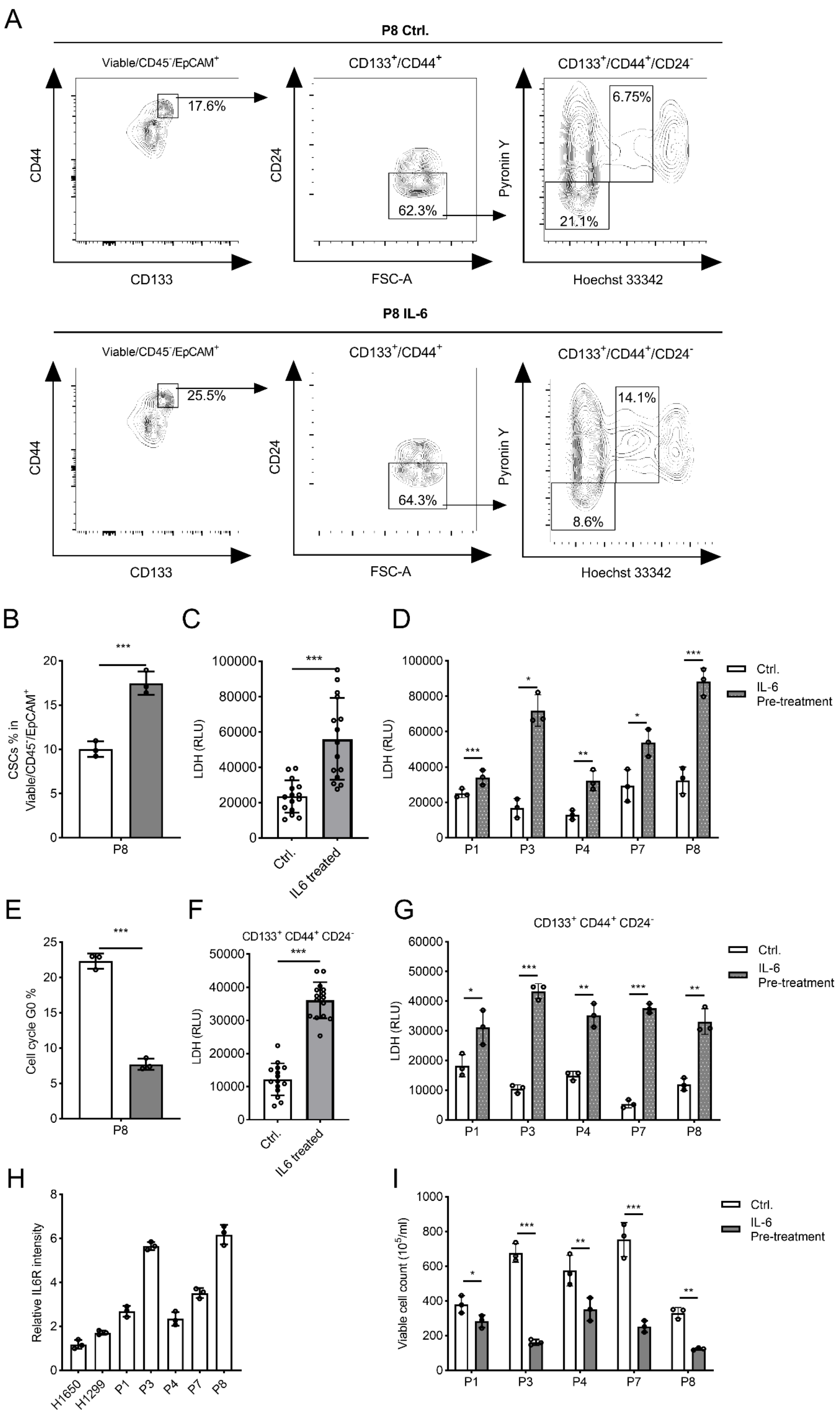
A
P8 Ctrl.
Viable/CD45⁻/EpCAM⁺
17.6%
CD44
CD133
CD133⁺/CD44⁺
CD24
62.3%
FSC-A
CD133⁺/CD44⁺/CD24⁻
6.75%
21.1%
Pyronin Y
Hoechst 33342
P8 IL-6
25.5%
64.3%
14.1%
8.6%
B
CSCs % in Viable/CD45⁻/EpCAM⁺
P8
C
LDH (RLU)
Ctrl.
IL6 treated
D
Ctrl.
IL-6 Pre-treatment
P1
P3
P4
P7
P8
E
Cell cycle G0 %
F
CD133⁺ CD44⁺ CD24⁻
G
CD133⁺ CD44⁺ CD24⁻
H
Relative IL6R intensity
H1650
H1299
I
Viable cell count (10⁵/ml)

Figure S5, IL6 pre-treatment significantly increases chemosensitivity in lung cancer

After IL6 pre-treatment with or without IL6 for 48 hours, five patients derived primary LUAD cells were seeded in same number and density (1×10^6 per 12 well plate well), and the cancer stem cells were sorted and seeded in 96 well plate (1×10^5 per well) followed by cytotoxic cis-diammine-dichloridoplatinum [II] (cDDP) treatment (5 μM) for 24 hours. After that the supernatants were collected for LDH measurement.

A-B & E, experimental setting, primary lung cancer cells originated from P8 were introduced, the cancer stem cell and the cell cycle FACS indicated IL6 pre-treatment significantly expanded the CSC population and promoted the entry into cell cycle.

C & D, the supernatant LDH level from primary lung cancer cells after cDDP chemotherapy, compared with control group, IL6 pre-treatment significantly increased the supernatant LDH cross all five primary lung cancer cells

F & G, the supernatant LDH level from cancer stem cells after cDDP chemotherapy, compared with control group the supernatant LDH levels were significantly elevated in IL6 pre-treated group.

H, the relative IL6 receptor level of all primary lung cancer cells and two lung cancer cell lines (H1299 and H1650), the intensity levels were standardized to IL6R level of H1650 cell line (set as 1). P3 and P8 have relative higher IL6R level, and they were also sensitive to the IL6 pre-treated cDDP chemotherapy.

I, viable cell counts of primary lung cancer cells after cDDP chemotherapy, compared with control group, IL6 pre-treatment significantly decreased the viable cell number cross all five primary lung cancer cells

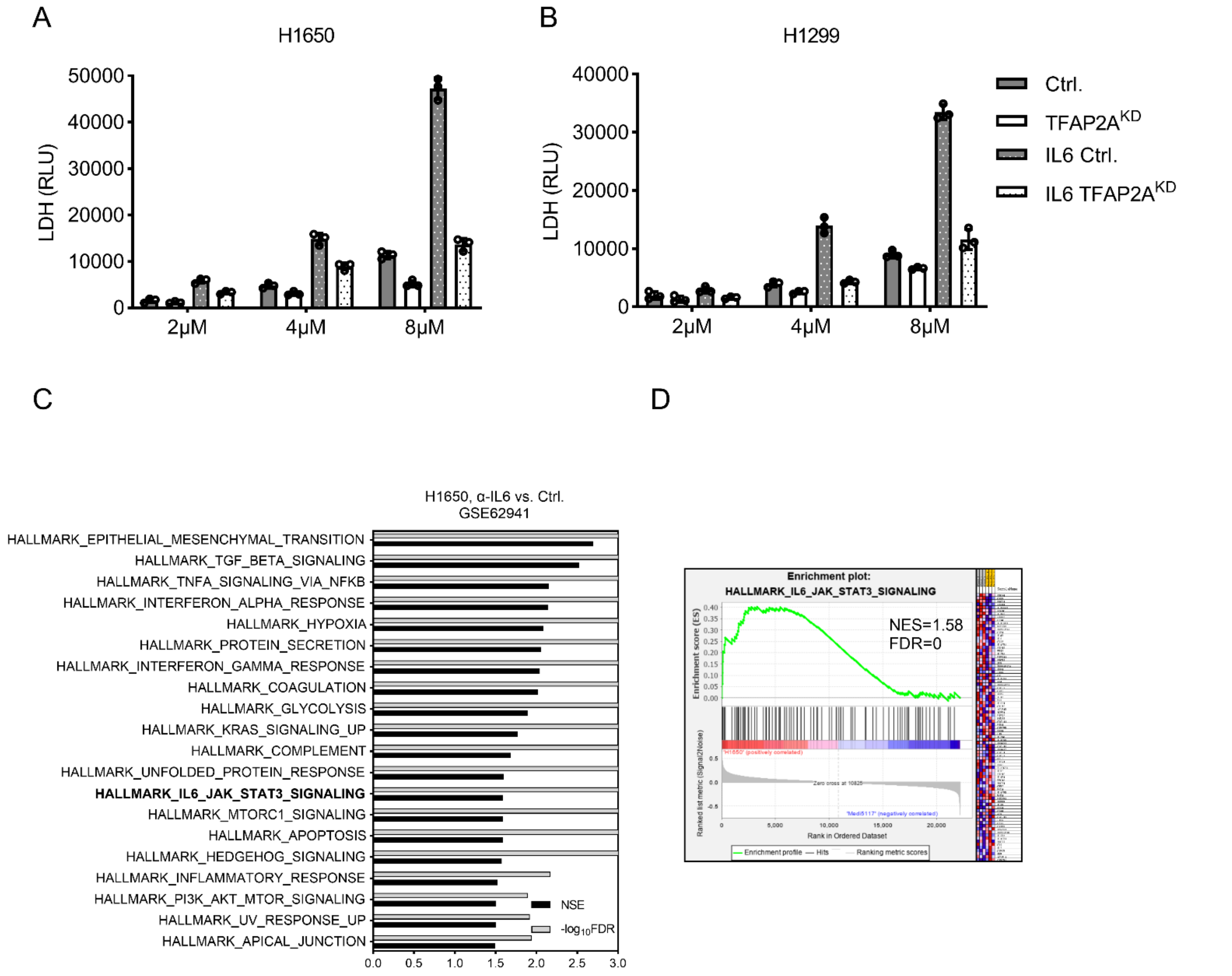

Figure S6, IL6 pre-treatment induced cDDP chemosensitivity showed an AP2α dependent manner

The AP2α knockdown (TFAP2A^{KD}) lung cancer cell lines (H1299 and H1650) were introduced. After IL6 pre-treatment for 48 hours, lung cancer cells were collected for cytotoxic cis-diammine-dichloridoplatinum [II] (cDDP) treatment in serial concentration for 24 hours, and the supernatant LDH levels were measured.

A & B, the supernatant LDH level in lung cancer cells after cDDP treatment for 24 hours. Dose dependent phenotypes were observed in cDDP *in-vitro* models, 8μM cDDP chemotherapy induced most cell death, IL6 pre-treatment significantly increased the cDDP induced cell death while TFAP2A^{KD} undermined the cDDP induced cell death, moreover, TFAP2A^{KD} significantly decreased the effects of IL6 pre-treatment.

C, open access GEO dataset GSM62941 (three anti-IL6 vs. three control H1650 cells) was submitted for the Gene Set Enrichment Analysis (GSEA), the listed pathways were influenced by anti-IL6 treatment. And the enrichment plot of IL6-JAK-STAT3 pathway, Normalized Enrichment Score (NES)=1.59, FDR q-value=0.

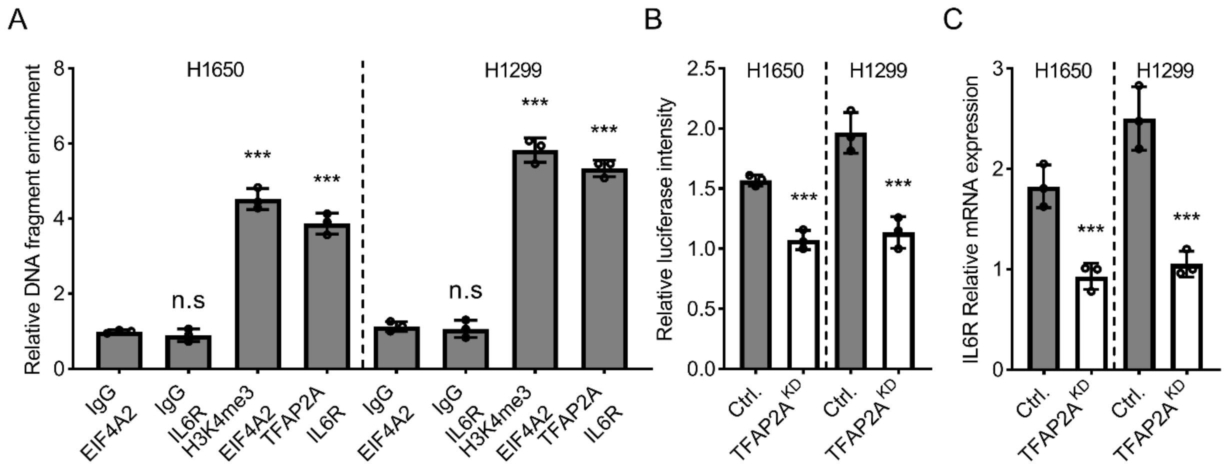

Figure S7, AP2α (TFAP2A) facilitates the activation of IL6-STAT3 signaling via promoting the IL6R expression

A, the ChIP-qPCR were performed by detecting the IL6R promotor region DNA fragments enriched by TFAP2A pulldown, compared to negative Ctrl. groups, our data indicated that, TFAP2A binds to the IL6R promotor region.

B & C, the duo-luciferase reporter assay and qRT-PCR were performed, reporter plasmids (with firefly luciferase) containing the promotor region of IL6R were introduced together with a background *Renilla* luciferase reporter plasmid. Our data indicated that, TFAP2A activated the IL6R expression transcriptionally.

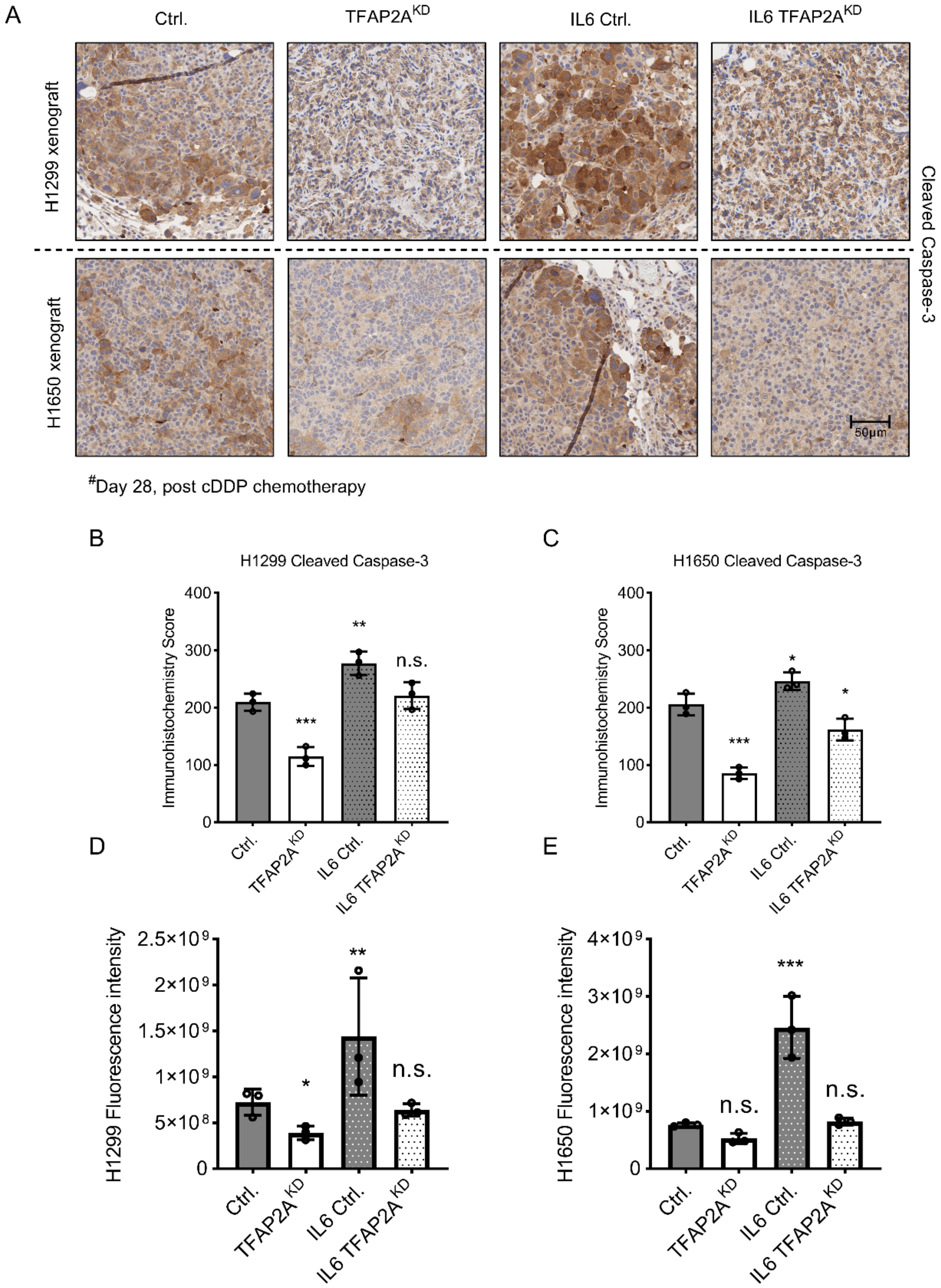

Figure S8, IL6 pre-treatment attenuates chemoresistance *in-vivo*

A, at day 28, the mice lungs were harvested for IHC staining, the cleaved caspase 3 was stained for evaluating apoptosis. Linked to Figure 2D.

B & C, the IHC score for cleaved caspase 3 in H1299 and H1650 xenograft mice lung. IL6 pre-treatment induced the most apoptosis while $TFAP2A^{KD}$ significantly undermined the chemosensitivity resulting less apoptosis.

D & E, the bioluminescence image of mice at day 20.

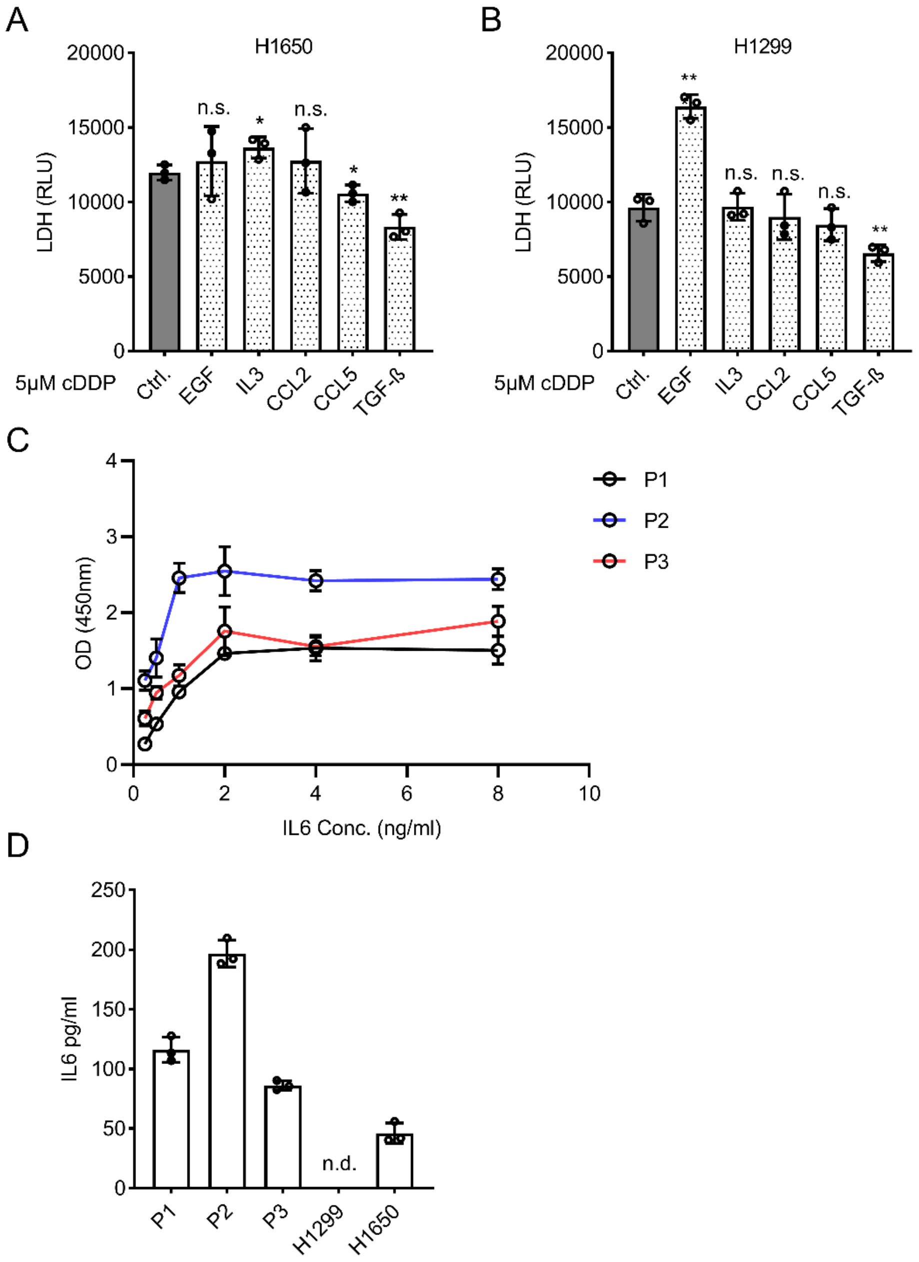

Figure S9, the impact of other cytokines pre-treatment on chemosensitivity

A & B, other cytokines including EGF (1 ng/ml), IL3 (10 ng/ml), CCL2 (20 ng/ml), CCL5 (50 ng/ml) and TGF-ß (1 ng/ml) were introduced for the pre-treatment assay. After cytokine pre-treatment for 48 hours, fresh medium was changed and cDDP was added in the medium with 8μM final concentration for 24 hours, after that the supernatants were collected for LDH measurements. The LDH level in the supernatant from H1650 and H1299 cells. Compared to the control groups, in H1650 cells IL3 pre-treatment slightly increased the chemosensitivity while CCL5 and TGF-ß pre-treatment decreased the chemosensitivity. And only in H1299 cells, the EGF pre-treatment significantly increased the chemosensitivity. Of note, in both H1650 and H1299 cells, TGF-ß pre-treatment significantly undermined the chemosensitivity.

C, to determine the concentration for IL6 treatment, three primary lung cancer cells (P1, P2 and P3) were introduced, after 48 hours of IL6 treatment in serial concentration the cell proliferation were evaluated by using CCK8 kit.

D, the supernatant IL6 level at baseline (n.d. for non-detectable), the data indicated that, our experimental setting was much higher compared to the natural supernatant IL6 level.

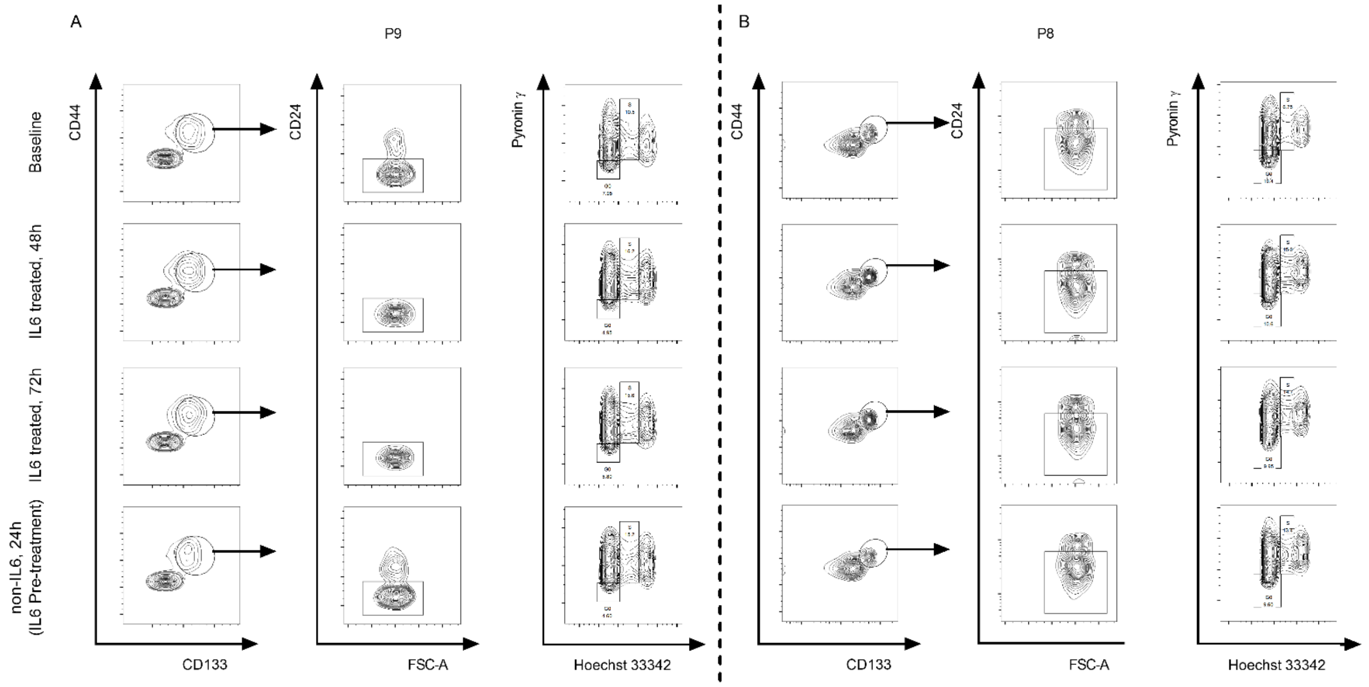

Figure S10, FACS plots P8 and P9

A, the FACS plots for cancer stem cells measurement and cell cycle distribution of patient derived lung cancer cells (patient sample 8 and 9), data in Figure 1E-G.

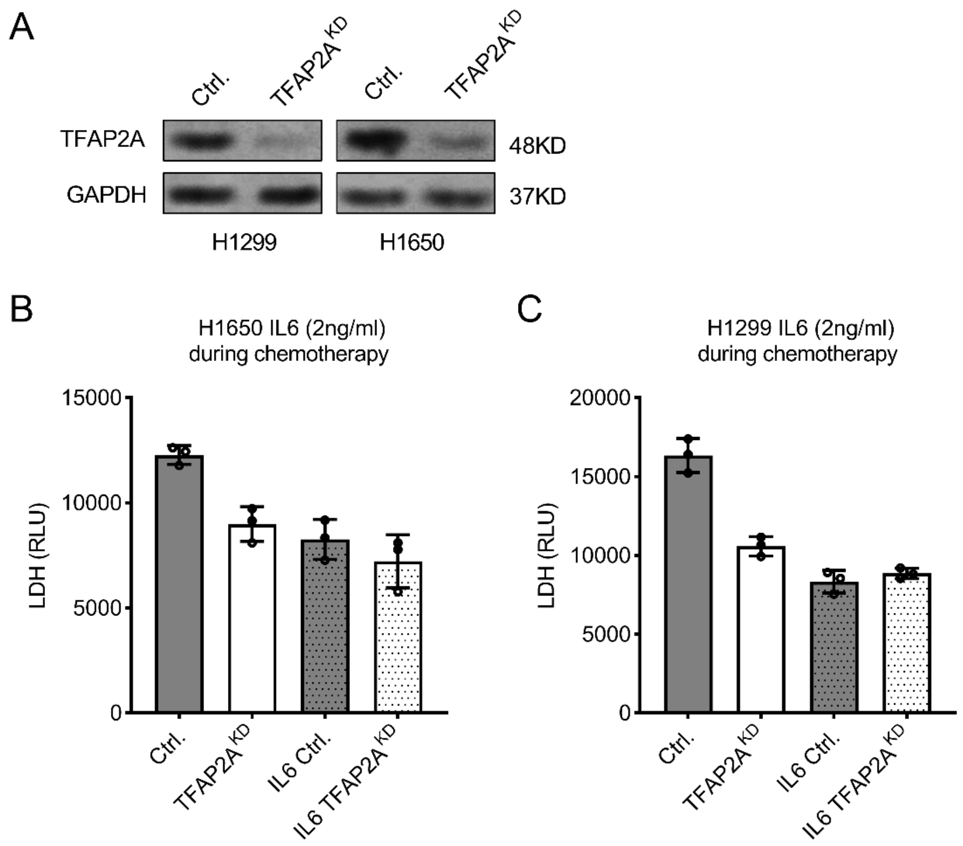

Figure S11, continues IL6 treatment contributes to the chemo-resistant

A, the western blot of TFAP2A, the TFAP2A knockdown could significantly decrease the TFAP2A level.
B & C, 2 ng/ml IL6 were added to the H1650 and H1299 cell culture medium during cDDP (8 μM) chemosensitivity, different to the IL6 pre-treatment assay, both IL6 treatment and $TFAP2A^{KD}$ significantly undermined the chemosensitivity in H1650 and H1299 cells.

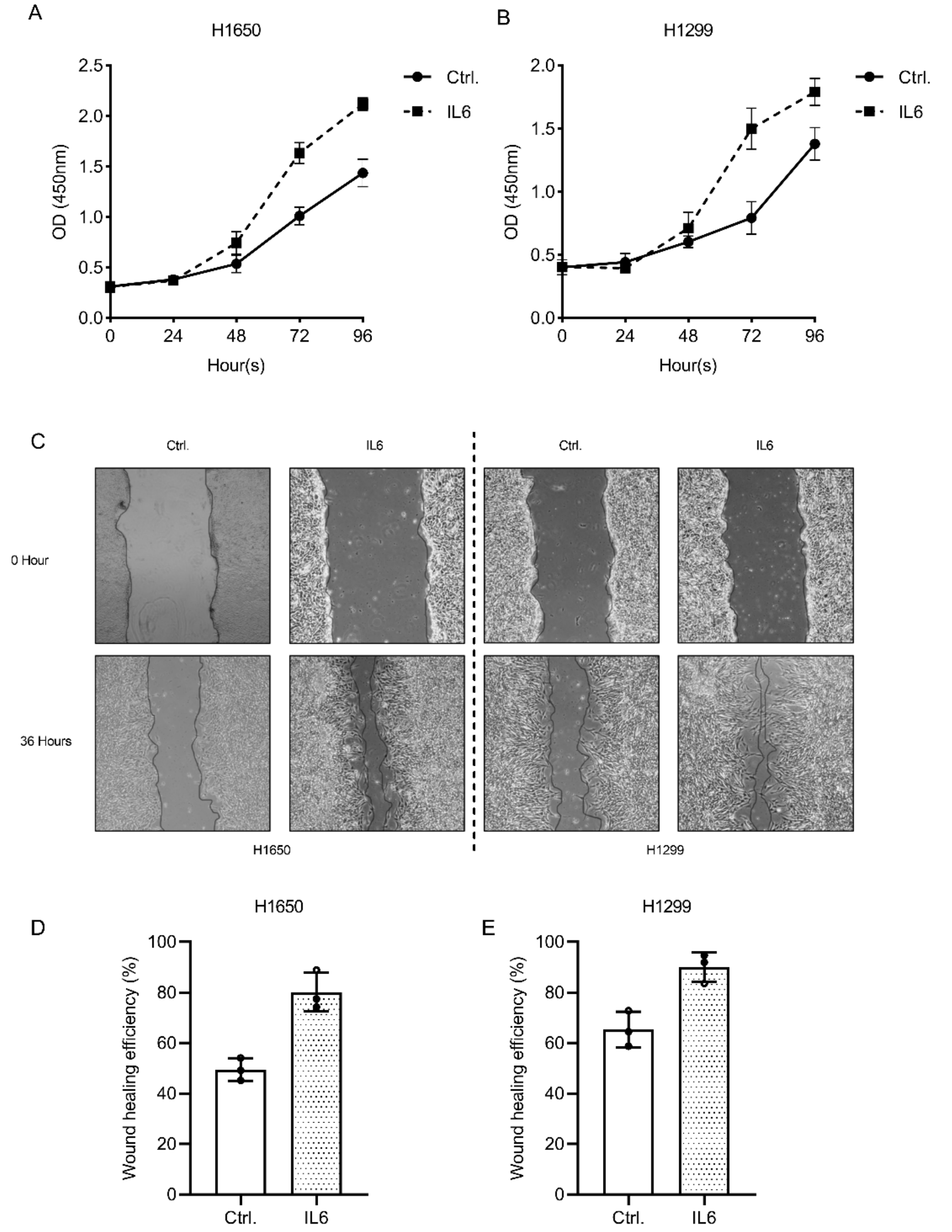

Figure S12, IL6 promotes cell proliferation and migration

A & B, H1650 and H1299 cells were treated with or without IL6 (2 ng/ml) for 96 hours, the cell proliferation rates were evaluated by cell counting kit 8 (CCK8) assay, and the supernatant were also collected for LDH measurements, but LDH were not detectable. The IL6 treated cells have advantage in proliferation.

C, H1650 and H1299 cells were treated with or without IL6 (2 ng/ml), the cell migration abilities were evaluated by wound healing assay.

D & E, the IL6 treated H1650 and H1299 cells have the advantage for migration.

Figure 1P H1299 p-STAT3

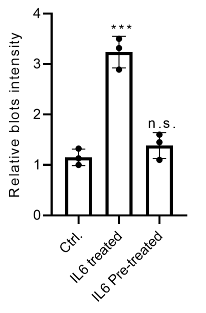

Figure 1P H1650 p-STAT3

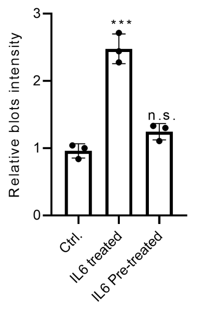

Figure 1P H1299 STAT3

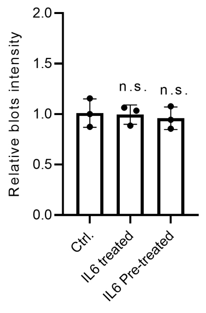

Figure 1P H1650 STAT3

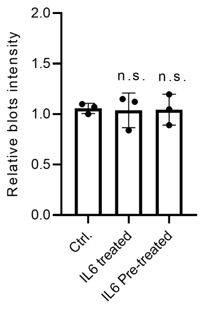

Figure 1P H1299 CCND1

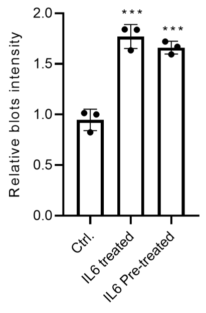

Figure 1P H1650 CCND1

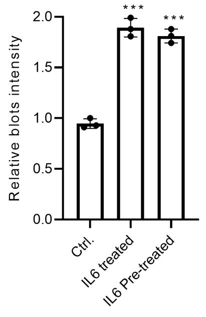

Figure 2B H1650 p-STAT3

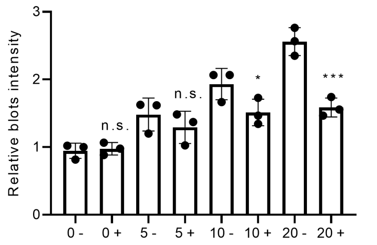

Figure 2B H1299 p-STAT3

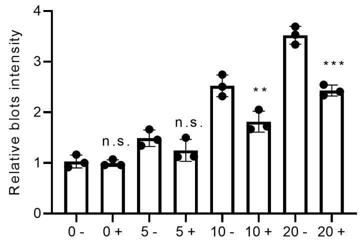

Figure 2B H1650 STAT3

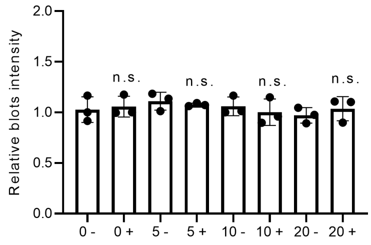

Figure 2B H1299 STAT3

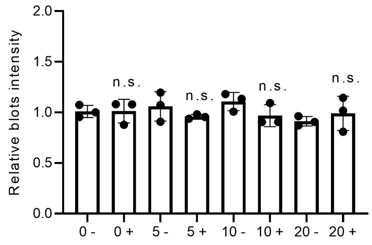

Figure 2B H1650 IL6R

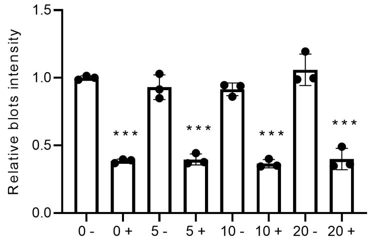

Figure 2B H1299 IL6R

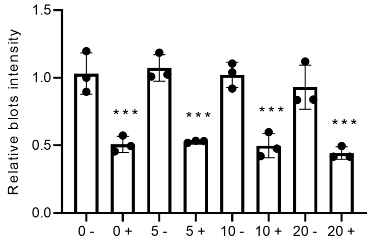

Figure 2B H1650 CCND1

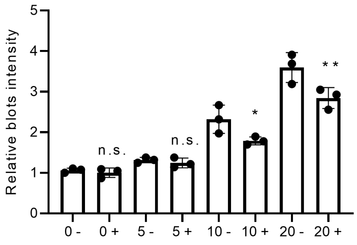

Figure 2B H1299 CCND1

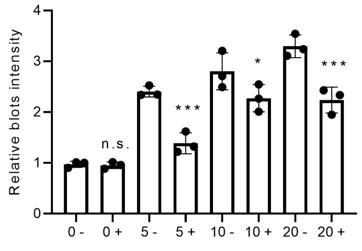

Figure S11A H1299 TFAP2A

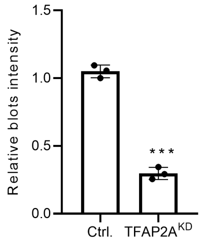

Figure S11A H1650 TFAP2A

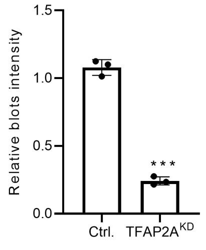

Figure S13, the quantification of immunoblot

The relative intensity of immunoblot was analyzed by ImageJ.
